# Specific cytokines in the inflammatory cytokine storm of patients with COVID-19-associated acute respiratory distress syndrome and extrapulmonary multiple-organ dysfunction

**DOI:** 10.1186/s12985-021-01588-y

**Published:** 2021-06-04

**Authors:** Jiajia Wang, Xinjing Yang, Yongsheng Li, Jian-an Huang, Junhong Jiang, Nan Su

**Affiliations:** 1 Department of Pulmonary and Critical Care Medicine, The First Affiliated Hospital of Soochow University, Pinghai Road No. 899, Suzhou, 215000 China; 2 Department of Emergency and Critical Care Medicine, The First Affiliated Hospital of Soochow University, Suzhou, 215000 China; 3 Department of Intensive Care Medicine, Tongji Hospital, Tongji Medical College, Huazhong University of Science and Technology, Wuhan, 430030 China; 4 Department of Pulmonary and Critical Care Medicine, Dushu Lake Hospital, Affiliated to Soochow University, Chongwen Road No. 9, Suzhou, 215000 China

**Keywords:** Inflammatory cytokine, SARS-CoV-2, COVID-19, Acute respiratory distress syndrome, Disseminated intravascular coagulation, Multiple-organ dysfunction

## Abstract

**Background:**

To date, specific cytokines associated with development of acute respiratory distress syndrome (ARDS) and extrapulmonary multiple organ dysfunction (MOD) in COVID-19 patients have not been systematically described. We determined the levels of inflammatory cytokines in patients with COVID-19 and their relationships with ARDS and extrapulmonary MOD.

**Methods:**

The clinical and laboratory data of 94 COVID-19 patients with and without ARDS were analyzed. The levels of inflammatory cytokines (interleukin 6 [IL-6], IL-8, IL-10, and tumor necrosis factor α [TNF-α]) were measured on days 1, 3, and 5 following admission. Seventeen healthy volunteers were recruited as controls. Correlations in the levels of inflammatory cytokines with clinical and laboratory variables were analyzed, furthermore, we also explored the relationships of different cytokines with ARDS and extrapulmonary MOD.

**Results:**

The ARDS group had higher serum levels of all 4 inflammatory cytokines than the controls, and these levels steadily increased after admission. The ARDS group also had higher levels of IL-6, IL-8, and IL-10 than the non-ARDS group, and the levels of these cytokines correlated significantly with coagulation parameters and disseminated intravascular coagulation (DIC). The levels of IL-6 and TNF-α correlated with the levels of creatinine and urea nitrogen, and were also higher in ARDS patients with acute kidney injury (AKI). All 4 inflammatory cytokines had negative correlations with PaO_2_/FiO_2_. IL-6, IL-8, and TNF-α had positive correlations with the APACHE-II score. Relative to survivors, non-survivors had higher levels of IL-6 and IL-10 at admission, and increasing levels over time.

**Conclusions:**

The cytokine storm apparently contributed to the development of ARDS and extrapulmonary MOD in COVID-19 patients. The levels of IL-6, IL-8, and IL-10 correlated with DIC, and the levels of IL-6 and TNF-α were associated with AKI. Relative to survivors, patients who died within 28 days had increased levels of IL-6 and IL-10.

## Background

In December 2019, the first case of infection by the severe acute respiratory syndrome coronavirus 2 (SARS-CoV-2) was reported in Wuhan City, China. The novel disease, termed coronavirus disease 2019 (COVID-19), remains a significant worldwide threat to public health [1]. As of February 15, 2021, there were nearly 110 million people were infected worldwide, more than 2 million deaths (World Health Organization, https://www.who.int/), and the number of cases continues to increase. Some COVID-19 patients present with mild illness during the early stage of disease, but then develop severe pneumonia, acute respiratory distress syndrome (ARDS), and multiple organ dysfunction (MOD) within a few days.

ARDS was reported as a common complication of human coronavirus pneumonia, and was also a leading cause of death in previous epidemics caused by SARS-CoV-1 and Middle East respiratory syndrome coronavirus (MERS-CoV). In the current COVID-19 pandemic, the prevention and treatment of lung injury in patients with ARDS is an issue of great concern. Wu et al. reported that 41.8% of COVID-19 patients developed ARDS and that these patients had a mortality rate of 52.4% [2]. ARDS is a type of rapidly progressive respiratory failure characterized by diffuse alveolar damage that is often accompanied by apoptosis of alveolar epithelial cells, alveolar and interstitial pulmonary edema, inflammatory cell infiltration, and pulmonary microthrombosis. In patients with COVID-19, this fatal condition often begins with direct virus invasion of alveolar epithelial cells and manifests as an overactivation of immune responses. The excessive immune responses are characterized by overproduction and prolonged high levels of numerous cytokines, such as proinflammatory cytokines (interleukin 1β [IL-1β], IL-6, IL-8, and tumor necrosis factor α [TNF-α]) and anti-inflammatory cytokines (IL-4, IL-10). The proinflammatory cytokines are responsible for initiating an inflammatory response against various pathogen infection, and the anti-inflammatory cytokines are crucial for the inhibition of inflammatory reaction and preservation of organ function. Overproduction of proinflammatory and anti-inflammatory cytokines is closely associated with disease severity and poor prognosis [3].

Inflammatory cytokine storm plays a crucial role in the development of ARDS, and previous researchers reported its presence in epidemics caused by SARS-CoV-1 and MERS-CoV [4]. It has been revealed that patients with severe COVID-19 present higher levels of IL-2, IL-6, IL-7, IL-10, and TNF-α than the patients with mild and moderate disease [5–7]. Compared with survivors, the level of IL-6 increased significantly in non-survivors, and it was considered as a predictor of mortality in COVID-19 patients [8, 9]. There is increasing evidence that the inflammatory cytokine storm in COVID-19 patients contributes to the development of ARDS, is associated with extrapulmonary MOD, and plays a decisive role in disease progression [10, 11].

However, specific cytokines associated with development of ARDS and extrapulmonary MOD have not been systematically reported. Here, we determined the levels and dynamic changes of multiple inflammatory cytokines in COVID-19 patients with ARDS, and then examined the relationships of different cytokines with extrapulmonary MOD.

## Materials and methods

### Subjects and data collection

**Fig. 1:**
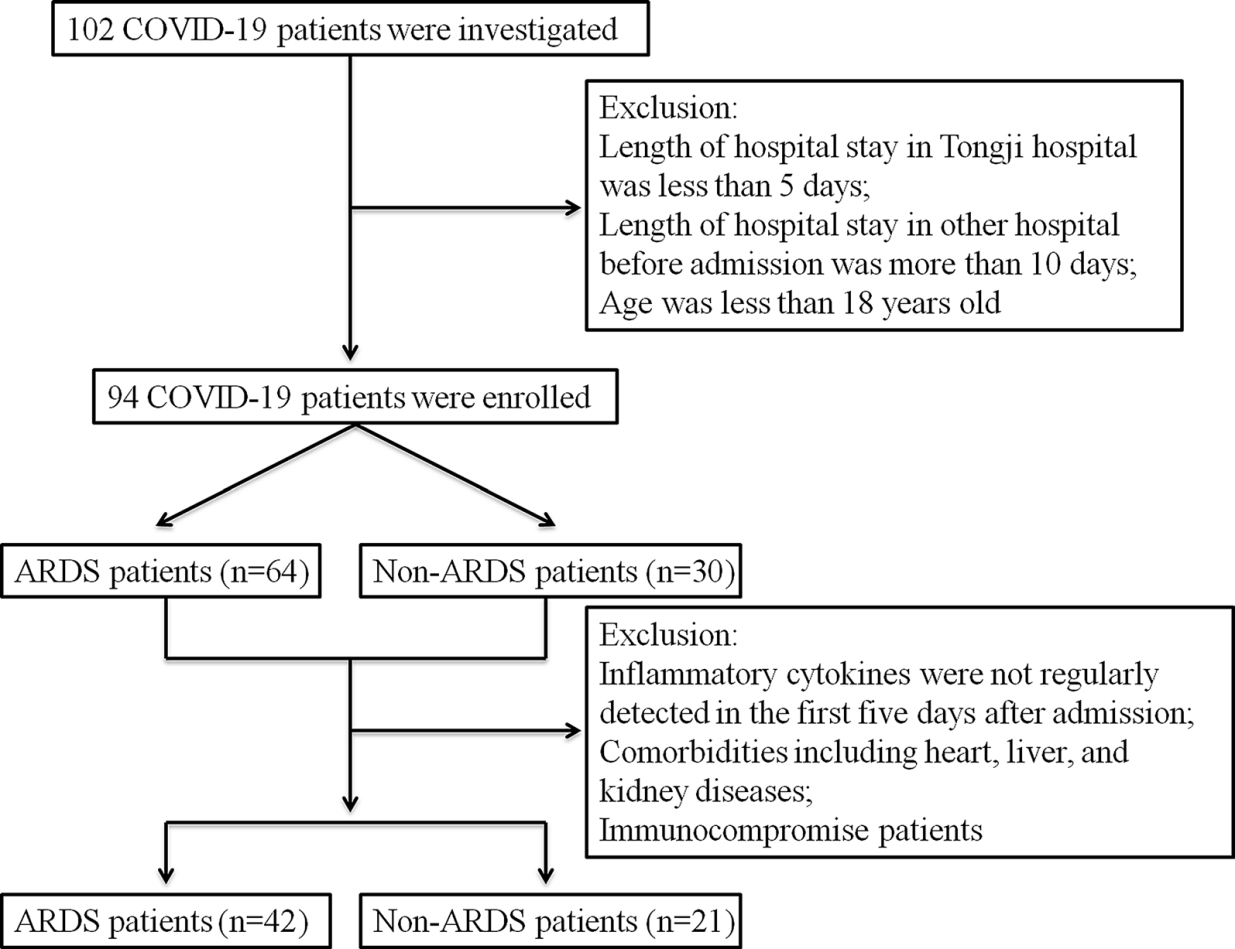
Disposition of 102 patients who were admitted with COVID-19 from February 1 to March 10, 2020

We retrospectively investigated 102 patients with COVID-19 who were admitted to Tongji Hospital of Huazhong University of Science and Technology from February 1, 2020 to March 10, 2020 (Fig. 1). The diagnosis of COVID-19 was according to World Health Organization interim guidelines and was confirmed by a positive result for SARS-CoV-2 RNA based on a PCR test (BGI Genomics Co. Ltd. Shenzhen, China). After exclusion of patients who had short hospital stays, stayed at another hospital for more than 10 days, or were under 18 years old, there were 94 eligible patients. These patients were divided into two groups: patients complicated with ARDS (n = 64) and patients without ARDS (n = 30). A total of 22 ARDS patients and 9 non-ARDS patients who had incomplete data for cytokine measurements, certain comorbidities, or were immunocompromised were excluded from some of the analyses. In addition, 17 adult volunteers without any underlying diseases were also enrolled as healthy controls. All symptoms, vital signs, laboratory tests, treatments, and clinical outcomes were recorded and analyzed. The severity of respiratory failure was assessed using the ratio of the partial pressure of arterial oxygen and the concentration of inspired oxygen (PaO_2_/FiO_2_). The severity of illness was evaluated using the Acute Physiology and Chronic Health Evaluation-II (APACHE-II) score and the Sepsis-related Organ Failure Assessment (SOFA) score.

### Definitions and diagnoses of ARDS, extrapulmonary MOD, and other conditions

The diagnosis of ARDS was according to the Berlin definition [12]. Cardiac dysfunction was determined as previously described for critically ill patients with influenza A virus infection, in which changes in the echocardiogram or electrocardiogram were coupled with an elevation of cardiac biomarkers [13]. The novel sepsis-3 definition was used to define septic shock [14]. Acute kidney injury (AKI) was defined using the Kidney Disease: Improving Global Outcomes (KDIGO) criteria [15]. Hepatic injury was defined according to elevation of bilirubin and aminotransferase as we had reported [16]. Disseminated intravascular coagulation (DIC) was defined by the New Chinese Diagnostic Scoring System for Disseminated Intravascular Coagulation [17]. The diagnosis of ARDS and extrapulmonary MOD was made during any period of hospital admission.

### Blood collection and cytokine measurements

The peripheral blood of all patients was collected at ICU admission (day-1), and on day-3 and day-5. Cytokines were measured in serum using specific kits that employed a chemiluminescent immunometric assay and an electrochemiluminescence method (DiaSorin Ltd., Vercelli, Italy and Roche Diagnostics Ltd., Germany).

### Statistical analysis

Means and standard deviations were used for descriptions of data with normal distributions, and medians with interquartile ranges (IQRs) for data with non-normal distributions. The two groups were compared using the independent samples *t*-test or the Mann–Whitney test, and those of several groups using Kruskal–Wallis test. Because the levels of cytokines had non-normal distributions in our patient population, the Spearman correlation coefficient (r) was used to assess their associations with different indices. A *p* value less than 0.05 was considered statistically significant, and asterisks were used to indicate the level of significance (*p < 0.05, **p < 0.01, ***p < 0.001). Changes of median cytokine levels during the first 5 days after ICU admission were used to characterize their dynamic changes. All statistical analyses were performed using SPSS version 22.0 and results were visualized using GraphPad Prism 5.0.

### Ethics approval

The Ethics Commission of Tongji Hospital of Huazhong University of Science and Technology approved this study, with a waiver of informed consent because the study was retrospective.

## Results

### Demographic characteristics, clinical data, disease indices, and laboratory markers in ARDS and non-ARDS patients

Table 1 shows the demographic characteristics, clinical data, and disease indices of COVID-19 patient in the ARDS and non-ARDS groups. The ARDS group was older (66.83 ± 10.93 vs. 59.8 ± 15.05 years, *p* = 0.012) and had more patients who were older than 65 years (59.4% vs. 36.7%, *p* = 0.04). Additionally, the ARDS group had a lower PaO_2_/FiO_2_, a higher APACHE-II score, and a higher SOFA score (all *p* < 0.05).

**Table 1:** Characteristics of COVID-19 patients at admission who did or did not develop ARDS

Variable	ARDS (n = 64)	non-ARDS (n = 30)	*P* value
Age (years)	66.83 ± 10.93	59.8 ± 15.05	0.012
Age (≥ 65 years)	38 (59.4%)	11 (36.7%)	0.04
Sex (male)	31 (48.4%)	16 (53.5%)	0.658
Current smoking	6 (9.4%)	4 (13.3%)	0.852
Underlying disease			
Hypertension	32 (50%)	11 (36.7%)	0.226
Diabetes	10 (15.6%)	7 (23.3%)	0.365
Heart disease	10 (15.6%)	3 (10%)	0.677
Liver disease	1 (1.6%)	0 (0%)	1
Kidney disease	1 (1.6%)	0 (0%)	1
Chronic lung disease	6 (9.4%)	1 (3.3%)	0.536
Malignancy	4 (6.3%)	1 (3.3%)	0.925
Immunocompromised	1 (1.6%)	0 (0%)	1
Ventilatory support			
HFNC	11 (17.5%)	3 (10%)	0.528
NIV	14 (22.2%)	4 (13.3%)	0.31
IV	8 (12.5%)	0 (0%)	0.104
Prior glucocorticoid treatment	32 (50%)	16 (53.3%)	0.763
Prior antibiotic treatment	41 (64.1%)	21 (70%)	0.571
Prior antiviral treatment	42 (65.6%)	20 (66.7%)	0.921
Days from onset to admission	11.70 ± 7.68	12.77 ± 7.09	0.523
PaO_2_/FiO_2_ (mmHg)	164.57 ± 75.41	267.13 ± 52.92	< 0.001
APACHE-II score	13.14 ± 4.23	8.84 ± 1.41	< 0.001
SOFA score	8.12 ± 2.59	5.88 ± 1.45	0.014

Data are presented as mean ± SD or n (%)

*HFNC* high-flow nasal cannula, *NIV* non-invasive ventilation, *IV* invasive ventilation, *APACHE-II* acute physiology and chronic health evaluation-II, *SOFA* sepsis-related organ failure assessment

The ARDS and non-ARDS groups also differed in many laboratory markers (Table 2). In particular, the ARDS group had significantly higher levels of white blood cells, neutrophils, incidence of lymphopenia, total bilirubin (TBIL), lactate dehydrogenase (LDH), blood urea nitrogen (BUN), highly sensitive C-reactive protein (hsCRP), procalcitonin (PCT), D-dimer, and prothrombin time (PT), and significantly lower levels of lymphocytes and albumin (all *p* < 0.05). These results are consistent with the more serious clinical condition of patients with ARDS.

**Table 2:** Laboratory indices of COVID-19 patients at admission who did or did not develop ARDS

Index	ARDS (n = 64)	Non-ARDS (n = 30)	*P* value
*Blood counts*			
White blood cells, × 10^9^/L	9.73 ± 5.42	5.31 ± 1.97	< 0.001
Neutrophils, × 10^9^/L	8.456 ± 5.15	3.99 ± 1.80	< 0.001
Monocytes, × 10^9^/L	0.52 ± 0.42	0.39 ± 0.21	0.070
Lymphocytes, × 10^9^/L	0.76 ± 0.58	1.05 ± 0.43	0.016
Platelets, × 10^9^/L	201.41 ± 120.11	205.4 ± 82.80	0.852
Lymphopenia, n (%)	55 (85.9%)	19 (63.3%)	0.013
Thrombocytopenia, n (%)	20 (31.3%)	4 (13.3%)	0.063
Hemoglobin, g/L	124.13 ± 22.18	127.80 ± 19.15	0.437
*Serum biochemistry*			
ALT, U/L	36.59 ± 33.15	28.47 ± 23.30	0.117
AST, U/L	46.28 ± 25.78	35.17 ± 29.80	0.067
TBIL, µmol/L	15.73 ± 10.58	9.35 ± 4.13	0.041
LDH, U/L	511.19 ± 264.81	276.30 ± 127.25	< 0.001
Creatinine, µmol/L	105.40 ± 126.52	78.57 ± 36.10	0.258
Urea nitrogen, mmol/L	9.73 ± 7.23	4.96 ± 2.47	0.001
Albumin, g/L	31.68 ± 6.10	34.34 ± 3.18	0.027
*Inflammatory markers*			
ESR, mm/h	44.37 ± 26.92	35.93 ± 23.00	0.197
hsCRP, mg/L	105.96 ± 69.63	47.30 ± 40.59	< 0.001
PCT, ng/mL	0.85 ± 1.36	0.07 ± 0.05	< 0.001
*Coagulation markers*			
D-dimer, µg/mL	7.59 ± 8.07	1.42 ± 2.05	< 0.001
PT, s	16.39 ± 4.89	13.97 ± 0.85	< 0.001
APTT, s	45.86 ± 12.47	43.30 ± 8.13	0.309
Fib, g/L	5.42 ± 1.97	5.17 ± 0.89	0.401

Data are presented as mean ± SD or n (%)

*ALT* alanine aminotransferase, *AST* aspartate aminotransferase, *TBIL* total bilirubin, *LDH* lactate dehydrogenase, *ESR* erythrocyte sedimentation rate, *hsCRP* high-sensitivity C-reactive protein, *PCT* procalcitonin, *PT* prothrombin time, *APTT* activated partial prothrombin time, *Fib* fibrinogen

### Inflammatory cytokines and their dynamics in ARDS and non-ARDS patients

**Fig. 2:**
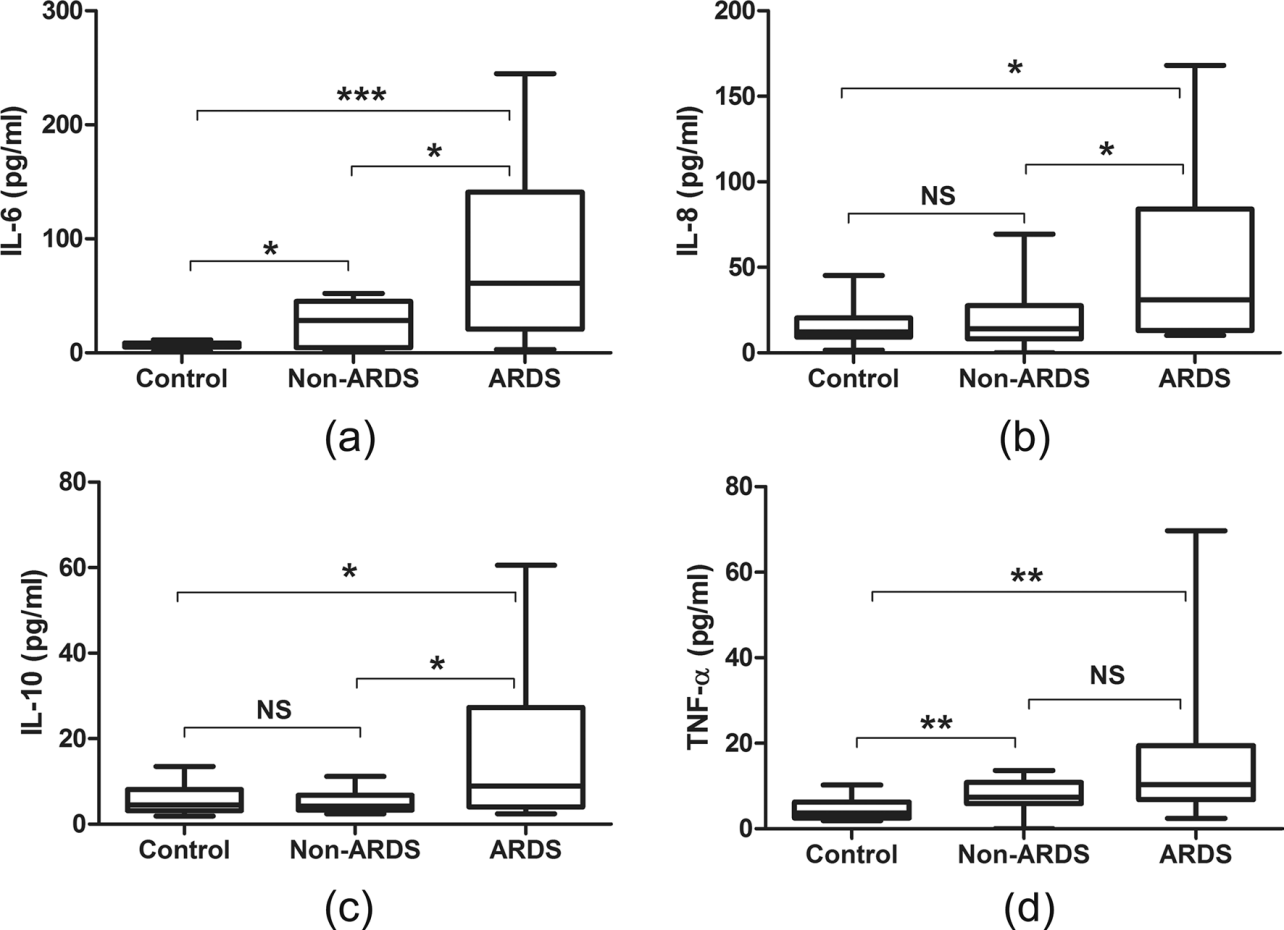
Levels of inflammatory cytokines in healthy controls and in COVID-19 patients at admission who did or did not develop ARDS. The levels of all 4 cytokines were significantly higher among ARDS patients than in the control group (**a**–**d**). The ARDS group had higher levels of IL-6, IL-8, and IL-10 than the non-ARDS group (**a**–**c**). Compared to control group, the non-ARDS group had higher levels of IL-6 and TNF-α (**a**, **d**). (*p < 0.05, **p < 0.01, ***p < 0.001)

We measured the levels of 4 inflammatory cytokines—IL-6, IL-8, IL-10, and TNF-α—in ARDS and non-ARDS patients upon ICU admission and in healthy controls (Fig. 2). Relative to the non-ARDS group, the ARDS group had higher levels of IL-6, IL-8, and IL-10 (all *P* < 0.05). Relative to the healthy controls, the non-ARDS group had higher levels of IL-6 and TNF-α (all *P* < 0.05). Notably, the levels of IL-8 and IL-10 were similar in the non-ARDS and control groups.

**Fig. 3:**
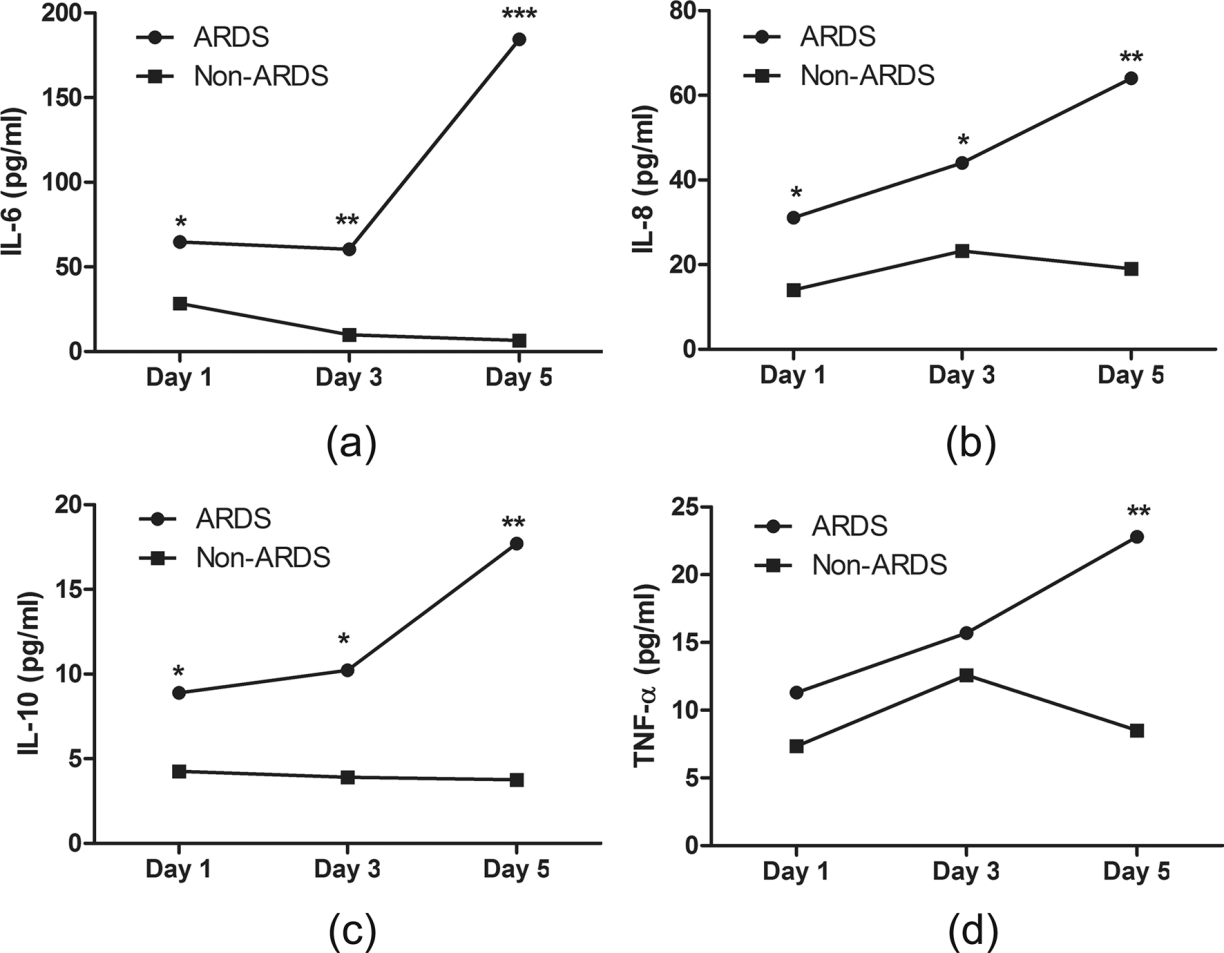
Dynamics of inflammatory cytokines in COVID-19 patients who did or did not develop ARDS. The ARDS group had progressive increases of all 4 cytokines after admission, while the non-ARDS group had only minor changes. (*p < 0.05, **p < 0.01, ***p < 0.001)

Our measurements of these 4 inflammatory cytokines at admission (day-1) and on day-3 and day-5 (Fig. 3) indicated the ARDS group had progressive increases of all 4 cytokines. In contrast, the non-ARDS group had only minor changes in the levels of IL-6, IL-8, and IL-10, and a transient elevation in the level of TNF-α.

### Correlations in the levels of inflammatory cytokines with clinical and laboratory data in ARDS patients

Our analysis of the correlations of the levels of cytokines with clinical data in patients with ARDS (Table 3) indicated that the IL-6 level had significant positive correlations with the levels PT, creatinine, BUN, cardiac troponin I (c-TnI), CK-MB, APACHE-II score, and SOFA score, and significant negative correlations with the levels of platelets, lymphocytes, and PaO_2_/FiO_2_. The IL-8 level had significant positive correlations with the levels of PT, APTT, and APACHE-II score, and a significant negative correlation with PaO_2_/FiO_2_. The IL-10 level had significant negative correlations with the levels of platelets, lymphocytes, and PaO_2_/FiO_2_. The TNF-α level had significant positive correlations with the levels of BUN and APACHE-II score, and a significant negative correlation with PaO_2_/FiO_2_.

**Table 3:** Correlations of the levels of inflammatory cytokines with clinical variables at admission of COVID-19 patients who developed ARDS (n = 42)

Clinical variable	IL-6	IL-8	IL-10	TNF-α
Neutrophils	r = − 0.027; *P* = 0.865	r = 0.019; *P* = 0.903	r = 0.261; *P* = 0.094	r = 0.003; *P* = 0.098
Platelets	r = − 0.409; *P* = 0.008	r = − 0.146; *P* = 0.364	r = − 0.388; *P* = 0.031	r = − 0.023; *P* = 0.884
Lymphocytes	r = − 0.408; *P* = 0.007	r = − 0.157; *P* = 0.320	r = − 0.337; *P* = 0.029	r = − 0.272; *P* = 0.082
Monocytes	r = − 0.272; *P* = 0.061	r = − 0.305; *P* = 0.05	r = − 0.192; *P* = 0.223	r = − 0.070; *P* = 0.658
PT	r = 0.322; *P* = 0.043	r = 0.351; *P* = 0.027	r = 0.26; *P* = 0.105	r = 0.128; *P* = 0.431
APTT	r = 0.193; *P* = 0.233	r = 0.355; *P* = 0.024	r = 0.241; *P* = 0.435	r = 0.132; *P* = 0.416
D-dimer	r = 0.001; *P* = 0.995	r = − 0.139; *P* = 0.399	r = − 0.045; *P* = 0.787	r = − 0.183; *P* = 0.265
Creatinine	r = 0.389; *P* = 0.012	r = 0.158; *P* = 0.313	r = 0.045; *P* = 0.772	r = 0.247; *P* = 0.098
Urea nitrogen	r = 0.356; *P* = 0.024	r = 0.287; *P* = 0.082	r = 0.157; *P* = 0.327	r = 0.312; *P* = 0.048
c-TnI	r = 0.356; *P* = 0.024	r = 0.013; *P* = 0.921	r = 0.165; *P* = 0.302	r = 0.035; *P* = 0.771
BNP	r = 0.215; *P* = 0.176	r = 0.153; *P* = 0.401	r = 0.187; *P* = 0.302	r = 0.014; *P* = 0.942
CK-MB	r = 0.425; *P* = 0.007	r = 0.025; *P* = 0.893	r = 0.015; *P* = 0.924	r = 0.127; *P* = 0.385
LDH	r = 0.267; *P* = 0.087	r = 0.105; *P* = 0.512	r = 0.274; *P* = 0.070	r = 0.058; *P* = 0.347
ALT	r = 0.082; *P* = 0.615	r = 0.257; *P* = 0.069	r = 0.211; *P* = 0.257	r = 0.154; *P* = 0.512
AST	r = 0.267; *P* = 0.075	r = 0.146; *P* = 0.366	r = 0.064; *P* = 0.707	r = 0.214; *P* = 0.172
TBIL	r = 0.254; *P* = 0.071	r = 0.142; *P* = 0.366	r = 0.118; *P* = 0.481	r = 0.064; *P* = 0.678
PaO_2_/FiO_2_	r = − 0.544; *P* < 0.001	r = − 0.321; *P* = 0.038	r = − 0.366; *P* = 0.017	r = − 0.436; *P* = 0.004
APACHE-II score	r = 0.660; *P* < 0.001	r = 0.329; *P* = 0.033	r = 0.26; *P* = 0.096	r = 0.446; *P* = 0.003
SOFA score	r = 0.465; *P* < 0.001	r = 0.128; *P* = 0.421	r = 0.168; *P* = 0.278	r = 0.292; *P* = 0.061

*PT* prothrombin time, *APTT* activated partial prothrombin time, *c-TnI* cardiac troponin I, *BNP* brain natriuretic peptide, *CK-MB* creatine kinase-isoenzyme MB, *LDH* lactate dehydrogenase, *ALT* alanine aminotransferase, *AST* aspartate aminotransferase, *TBIL* total bilirubin, *APACHE-II* acute physiology and chronic health evaluation-II, *SOFA* sepsis-related organ failure assessment

Our analysis of complications (Table 4) indicated the prevalences of septic shock, DIC, AKI, and cardiac injury were significantly greater in the ARDS group than in the non-ARDS group. Hepatic injury also had a higher prevalence in the ARDS group, but the difference was not significant.

**Table 4:** Prevalences of complications during hospitalization of COVID-19 patients in the ARDS and non-ARDS groups

Complication	Total (n = 94)	ARDS (n = 64)	Non-ARDS (n = 30)	*P* value^a^
Septic shock	51 (54.25%)	46 (71.87%)	5 (16.67%)	< 0.001
DIC	32 (34.04%)	28 (43.75%)	4 (13.33%)	0.008
AKI	36 (38.29%)	34 (53.12%)	2 (6.67%)	< 0.001
Cardiac injury	53 (56.38%)	50 (78.12%)	3 (10%)	< 0.001
Hepatic injuryy	21 (22.34%)	18 (28.13%)	3 (10%)	0.089
28-day survival	43 (45.74%)	18 (28.13%)	25 (83.33%)	< 0.001

^a^The comparison of the ARDS and non-ARDS groups

*DIC* disseminated intravascular coagulation, *AKI* acute kidney injury

We also determined the levels of inflammatory cytokines on admission in ARDS patients with stratification by clinical outcome (Table 5). The results indicated that patients who developed DIC had higher levels of IL-6, IL-8, and IL-10; patients who developed AKI had higher levels of IL-6 and TNF-α; and patients who died within 28 days of admission had higher levels of IL-6 and IL-10.

**Table 5:** Levels of inflammatory cytokines at admission in different subgroups of patients who developed ARDS (n = 42)*

Outcome	Subgroups	IL-6	*P* value	IL-8	*P* value	IL-10	*P* value	TNF-α	*P* value
Septic shock	Yes (n = 33)	54.88 (26.64–161.65)	0.29	21.90 (13.05–60.70)	0.417	10.50 (5.30–20.05)	0.127	10.3 (7.15–19.70)	0.391
	No (n = 9)	31.76 (19.30–72.91)		29.9 (20.20–72.10)		6.10 (4.25–13.35)		9.3 (7.6–10.7)	
DIC	Yes (n = 21)	96.99 (37.62–214.90)	< 0.001	47.00 (19.3–103.25)	0.016	14.40 (5.85–25.4)	0.006	11.0 (7.8–26.4)	0.131
	No (n = 21)	29.32 (11.23–47.98)		17.50 (11.95–30.45)		6.20 (5.0–9.85)		9.30 (7.15–12.35)	
AKI	Yes (n = 21)	59.83 (31.02–200.90)	0.013	30.50 (15.55–103.25)	0.199	10.70 (5.40–20.05)	0.087	11.30 (8.4–26.4)	0.047
	No (n = 21)	35.50 (18.11–106.09)		22.90 (12.75–55.20)		6.4 (5.0–13.20)		9.30 (6.85–10.9)	
Cardiac injury	Yes (n = 33)	59.83 (26.64–161.65)	0.112	30.30 (14.50–75.25)	0.567	10.7 (5.3–20.05)	0.112	10.30 (7.30–19.70)	0.171
	No (n = 9)	29.54 (19.30–47.98)		22.90 (15.45–48.55)		6.1 (4.23–11.0)		9.30 (6.9–9.77)	
Hepatic injuryy	Yes (n = 13)	64.78 (34.74–186.15)	0.268	30.30 (16.80–89.60)	0.419	10.08 (5.3–25.4)	0.332	11.30 (7.45–41.55)	0.257
	No (n = 29)	43.38 (20.98–99.99)		21.90 (13.70–68.45)		7.9 (5.12–15.0)		9.5 (7.5–13.05)	
28-day survival	No (n = 32)	60.46 (30.36–185.03)	0.018	30.01 (14.25–71.93)	0.432	10.8 (5.4–24.08)	0.014	10.30 (7.48–19.68)	0.247
	Yes (n = 10)	28.81 (10.12–47.55)		23.95 (16.48–42.50)		5.5 (4.19–8.18)		8.6 (6.45–10.50)	

^*^After exclusion of 22 patients, as indicated in Fig. 1

*DIC* disseminated intravascular coagulation, *AKI* acute kidney injury

### Dynamics of inflammatory cytokines in survivors and non-survivors

**Fig. 4:**
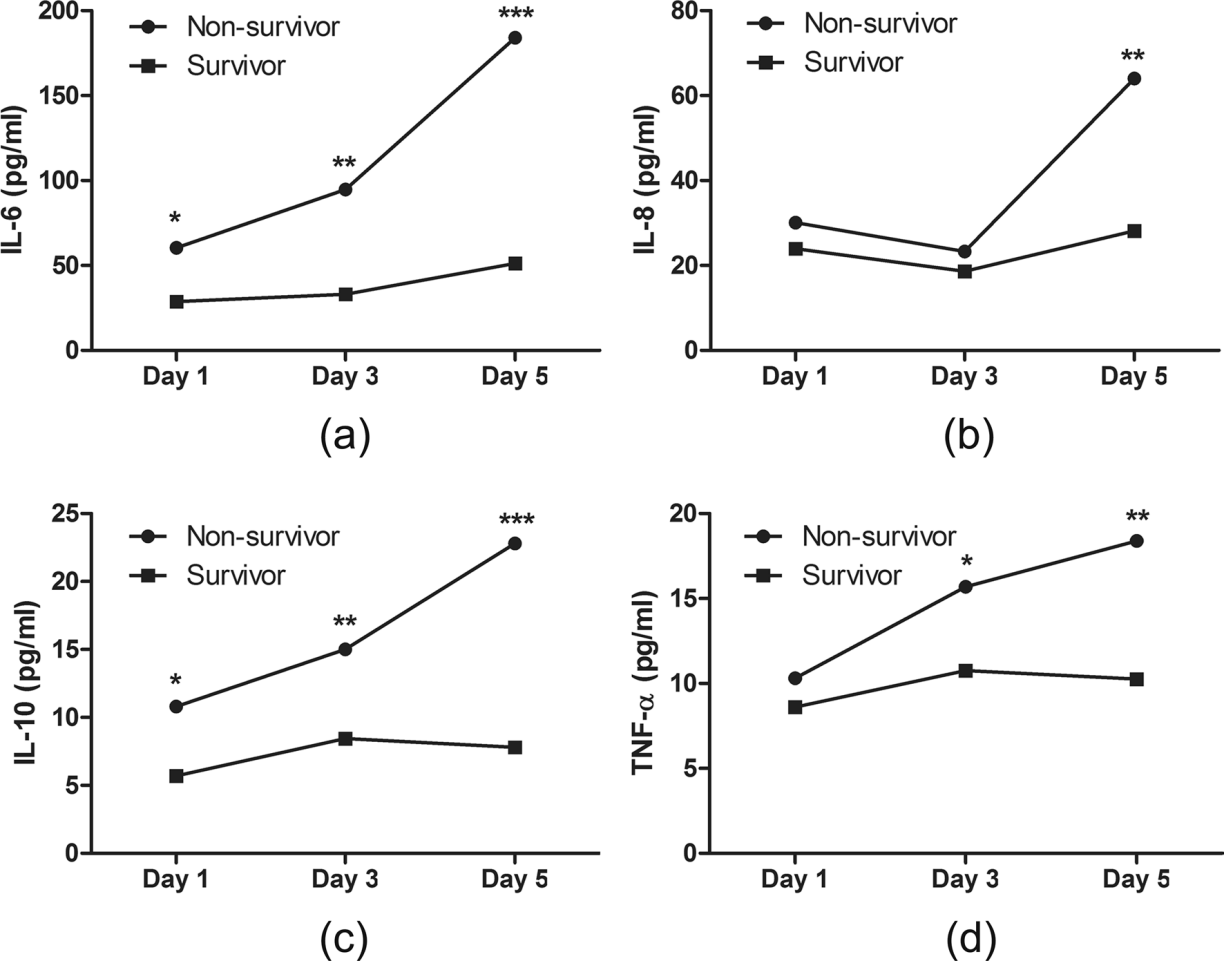
Dynamics of inflammatory cytokines in COVID-19 patients who did or did not die within 28 days after admission. The levels of all 4 cytokines in non-survivors increased significantly during the first 5 days after admission, while the survivors only showed small changes. (*p < 0.05, **p < 0.01, ***p < 0.001)

The levels of all 4 inflammatory cytokines in patients who survived for 28 days only had small changes during the first 5 days after ICU admission (Fig. 4). In contrast, the levels of these same cytokines increased significantly in non-survivors, especially the levels of IL-6 and IL-10, whose concentrations increased by more than twofold within 5 days.

## Discussion

This study systematically described the detailed characteristics of the inflammatory cytokine storm in patients with COVID-19-associated ARDS. Our study had five major findings. First, the levels of inflammatory cytokines at admission were higher in patients who developed ARDS than in healthy controls, and the levels of these cytokines continued to increase after admission. Second, the levels of IL-6, IL-8, and IL-10 at admission were higher in patients who developed ARDS than in those who did not develop ARDS, and these 3 cytokines were also associated with the presence of DIC. Third, higher levels of IL-6, IL-8, IL-10, and TNF-α at admission were associated with respiratory failure and disease deterioration. Fourth, the levels of IL-6 and TNF-α at admission were associated with the development of AKI. Fifth, IL-6 and IL-10 had higher levels at admission in patients who died within 28 days than in survivors, and these cytokines became increasingly elevated after admission of patients who subsequently died.

Previous research demonstrated that older age is a risk factor for the development of ARDS in patients with COVID-19, because the elderly often present with weakened immune systems [2]. In agreement, we also found that ARDS was more common in COVID-19 patients who were age 65 years and older. There is evidence that severe inflammatory cell infiltration, extensive release of cytokines culminating in a cytokine storm, and coagulation dysfunction contributed to the development of ARDS [2]. Compatible with this pathogenesis, we found that the levels of white blood cells, neutrophils, inflammatory markers (hsCRP and PCT), and coagulation parameters (D-dimer and PT) were significantly greater in patients who developed ARDS. Lymphopenia is another striking abnormality in COVID-19 patients, especially in those with ARDS, and is associated with poor prognosis [18]. A previous study of MERS-CoV reported that an inflammatory cytokine storm contributed to increased lymphocyte apoptosis [19].

Previous research concluded that the cytokine storm played a decisive role in the development of ARDS and MOD in COVID-19 patients [10]. In our study, relative to the healthy controls, the ARDS group had higher levels of all of the 4 studied cytokines, and the levels of these cytokines continued to increase after ICU admission in patients who developed ARDS. Comparison of the ARDS and non-ARDS groups indicated that the levels of IL-6, IL-8, and IL-10 at admission were significantly increased in the group that developed ARDS, suggesting that these three cytokines contributed to the pathogenesis of ARDS. Thus, monitoring the levels of multiple cytokines, especially IL-6, IL-8, and IL-10, during the early stages of COVID-19 may help to identify patients who have the greatest risk for development of ARDS. We found no differences in the levels of IL-8 and IL-10 between non-ARDS group and the healthy controls. A similar result was reported in a study of community-acquired pneumonia, and the authors considered these cytokines as specific indicators of severe disease [20]. Thus, our findings are consistent with this previous study.

ARDS is a leading cause of death in patients with viral pneumonia caused by SARS-CoV-1, MERS-CoV, and SARS-CoV-2 [21]. Actually, MOD secondary to ARDS is the main cause of death, because respiratory failure alone only accounted for 16% of deaths in patients with ARDS [22]. COVID-19 originates in the lungs, often as severe pneumonia and/or ARDS, and patients may then deteriorate into extrapulmonary organ dysfunction. The overproduction of inflammatory cytokines contributes to the development of microthrombosis and DIC in patients with severe COVID-19 pneumonia [10]. Emerging evidence indicates that patients with COVID-19 have a high risk for DIC, and previous studies reported this complication in 71.4% of non-survivors [23, 24].

However, no previous studies have examined the prevalence of DIC in COVID-19-associated ARDS patients or the prognosis of these patients. We found that the total prevalence of DIC among COVID-19 patients was 34.04%, and the prevalence was greater in the ARDS group than in the non-ARDS group (43.75% vs*.* 13.33%). Our analysis of non-survival at 28 days indicated that 58.82% of patients with COVID-19-associated ARDS developed DIC (data not shown). ARDS has a close relationship with DIC, as these two complications are each characterized by inflammatory cytokine-induced microthrombosis. Exaggerated inflammation and a dysregulated coagulation system thus mutually enhance the progression to ARDS and DIC. Very often, a patient with severe COVID-19 experiences an uncontrolled inflammatory response during ARDS, and this response functions as a trigger for the onset of DIC [25, 26]. No previous studies have examined the specific characteristics of the inflammatory cytokine storm in COVID-19 patients with ARDS and DIC. We found that the levels of IL-6, IL-8, and IL-10 in our study had significant correlations with pathological coagulation parameters, and were also greater in ARDS patients with DIC than those without DIC. Thus, these three cytokines may play an important role in the pathogenesis of DIC in patients with ARDS.

A previous multicenter observational study reported that AKI occurred in 44.3% of patients with ARDS [27]. AKI is a life-threatening clinical event that can occur during COVID-19, and the incidence ranges from 25 to 61.5% in these patients [28]. We found that the incidence of AKI in severe COVID-19 patients was 38.29% and that the incidence was 53.12% in COVID-19 patients with ARDS. The pathogenesis of AKI in COVID-19 appears to be complex and multifactorial. Currently, direct SARS-CoV-2 invasion of renal tubular cells and ischemic reperfusion injury are believed to contribute to AKI [29]. However, the systemic inflammatory response derived from injury of the lungs may also play an important role in the development of AKI, because the overproduction of inflammatory cytokines in ARDS is associated with AKI [30].

A previous study of influenza virus-induced ARDS reported that patients who developed AKI had higher levels of IL-6 and IL-8 and lower level of TNF-α [31]. We found that the levels of IL-6 and TNF-α were significantly increased in ARDS patients with AKI compared to patients without AKI, but the level of IL-8 was not significantly different between these two groups. These results suggest the possibility that IL-6 and TNF-α have specific functions in the development of AKI among patients with COVID-19-associated ARDS. Extrarespiratory organ dysfunction is common in patients with severe COVID-19, and previous research concluded the inflammatory cytokine storm contributed to this outcome [32]. Indeed, our patients had high incidences of cardiac injury (78.12%), hepatic injury (28.13%), and septic shock (71.87%). However, the levels of inflammatory cytokines in the subgroups with these different complications were not significantly different.

Previous studies confirmed that higher levels of inflammatory cytokines were associated with poorer prognosis in patients with ARDS. For example, Lee et al. studied patients with community acquired pneumonia (CAP)-induced ARDS and reported that relative to survivors, non-survivors had higher levels of IL-6, IL-8, and IL-1β in bronchoalveolar lavage fluid (BALF) and higher levels of IL-6 and IL-10 in peripheral blood samples [33]. Remarkably, the levels of serum IL-6 and IL-10 in our patients had the same trends described by Lee et al. [33]. Moreover, the levels of serum IL-6 and IL-10 in our non-survivors significantly increased following admission. Thus, the levels of specific cytokines at admission and their changes over time may provide important prognostic information for patients with COVID-19-associated ARDS.

Among the cytokines we measured, the levels of proinflammatory cytokine IL-6 and anti-inflammatory cytokine IL-10 at admission were significantly higher in patients who developed ARDS, and had a close relationship with indices of illness severity and 28-day survival. IL-6 and IL-10 are two essential components of inflammatory response in almost all infectious diseases released by a range of different cells. For example, IL-6 is produced by macrophages, lymphocytes, fibroblasts, and endothelial and epithelial cells, and IL-10 is secreted by dendritic cells, macrophages, T cells, natural killer cells, and B cells [34, 35]. The various cellular sources of IL-6 and IL-10 indicate complex and multiple effect on both pathogen and host. In present study, high IL-6 level is convinced of facilitating SARS-CoV-2 clearance, but inevitably causing tissue damage and even life-threating multiple organ failure during acute infection. The high IL-10 level could be protective in early stage of SARS-CoV-2 infection but become harmful as it suppresses inflammatory response and impedes pathogen clearance. The relative amounts of proinflammatory and anti-inflammatory cytokine production are supposed to be critical for safe resolution of infection, however, they are difficult to keep balance in such highly pathogenic viral infection.

Theoretically, anti-cytokine therapies and immunomodulators may be effective treatments to curb the excessive inflammatory response and ameliorate the subsequent injury. A recent study reported that administration of an interleukin-6 receptor antagonist (tocilizumab) to patients with severe COVID-19 provided a therapeutic benefit, and more patients are being enrolled in clinical trials testing the efficacy of this agent [36]. Moreover, other anticytokine agents, such anakinra, an IL-1 receptor antagonist, and anti-TNF-α agents also have potential as treatments for COVID-19 [37, 38]. IL-10 blockades revealed a capacity of enhancing pathogen clearance in previous mouse model, however, their application in clinical settings for COVID-19 patients has not been reported [39, 40].

Some limitations of this study should be mentioned. First, more than 50% of the patients took antiviral, antibiotic, and/or glucocorticoid before admission, and this could have affected their levels of cytokines measured at admission. Second, the total number of diabetes mellitus (DM) patients was only 17 in our study. The features of cytokine profiling and their association with MOD in DM patients should be further investigated in a larger cohort as hyperglycemia contributes to increased inflammatory response and enhanced disease severity in COVID-19 patients [41, 42]. Third, we did not measure cytokine levels in BALF, because this can be difficult, risky, and cannot be performed regularly due to the high pathogenicity of SARS-CoV-2. Fourth, our findings were from a single center in China and may not be representative of other populations. Despite these limitations, our findings that several serum cytokines were closely associated with the development of ARDS, extrapulmonary MOD, and patient prognosis have possible implications for the management of COVID-19 patients.

## Conclusion

In conclusion, we found that the inflammatory cytokine storm experienced by patients with severe COVID-19 was associated with ARDS, extrapulmonary MOD, and poor prognosis. Furthermore, elevated levels of certain specific cytokines during the early stage of COVID-19 may contribute to the subsequent dysfunction of different organs. ARDS is a product of the inflammatory cytokine storm following SARS-CoV-2 infection, and also exacerbates the inflammatory cytokine storm. Therefore, effective inhibition of the inflammatory cytokine storm during the early stages of COVID-19 may be an effective therapeutic strategy to prevent the development of ARDS, alleviate extrapulmonary MOD, and improve the prognosis of COVID-19 patients.

## Data Availability

The data in the current study are available from the corresponding author on reasonable request.

## Abbreviations

COVID-19: coronavirus disease 2019
ARDS: acute respiratory distress syndrome
MOD: multiple-organ dysfunction
DIC: disseminated intravascular coagulation
AKI: acute kidney injury
SARS-CoV-2: severe acute respiratory syndrome coronavirus 2
PaO_2_/FiO_2_: partial pressure of arterial oxygen and the concentration of inspired oxygen
APACHE-II: acute physiology and chronic health evaluation-II
SOFA: sepsis-related organ failure assessment
HFNC: high-flow nasal cannula
NIV: Non-invasive ventilation
IV: invasive ventilation
ALT: alanine aminotransferase
AST: aspartate aminotransferase
TBIL: total bilirubin
LDH: lactate dehydrogenase
ESR: erythrocyte sedimentation rate
hsCRP: high-sensitivity C-reactive protein
PCT: procalcitonin
PT: prothrombin time
APTT: activated partial prothrombin time
Fib: fibrinogen
c-TnI: cardiac troponin I
BNP: brain natriuretic peptide
CK-MB: creatine kinase-isoenzyme MB
